# Designing and Conducting Randomized Controlled Trials: Basic Concepts for Educating Early Researchers in the Field of Clinical Nutrition

**DOI:** 10.7759/cureus.17036

**Published:** 2021-08-09

**Authors:** Charu Arora, Anita Malhotra, Piyush Ranjan, Akshay Kumar

**Affiliations:** 1 Food and Nutrition, University of Delhi, New Delhi, IND; 2 Food and Nutrition, Lakshmibai College, University of Delhi, New Delhi, IND; 3 Medicine, All India Institute of Medical Sciences, New Delhi, IND; 4 Internal Medicine, All India Institute of Medical Sciences, New Delhi, IND

**Keywords:** clinical nutrition, dietitians, research design, randomized controlled trial, evidence based practice

## Abstract

Randomized controlled trials (RCTs) provide the best quality evidence to steer patient care in the field of clinical nutrition. However, designing and conducting an RCT, analyzing data, interpreting and reporting its findings is rather complex for young researchers working in the field of clinical nutrition. This review article attempts to educate early researchers by offering a simple step by step guide on planning the key aspects (randomization, allocation concealment, blinding, outcome measures) of a trial, and highlighting the practical considerations (ethical clearance, trial registry, patient recruitment, trial monitoring) to be kept in mind while conducting a trial contextualised to clinical nutrition settings.

## Introduction and background

Randomized controlled trials (RCTs) generate the best quality of evidence in clinical nutrition practice and play an instrumental role in validating any nutrition/lifestyle intervention [1]. The practice of evidence-based nutrition has created a huge demand for well-designed and systematically performed trials in the field of clinical nutrition.

Conducting nutrition and lifestyle intervention trials is challenging as it involves several interacting components and requires rigorous evaluation to assess the effectiveness of such trials [2]. Registered dietitians, postgraduate nutrition students, dietitians and young faculty in the field of clinical nutrition science often lack the desired training and exposure to conduct clinical trials. Moreover, they are burdened with a high load of indoor as well as outdoor patient counseling, leaving them little time to focus on designing and conducting research. The academic departments of Nutrition and Dietetics, especially in low and medium income countries, find it difficult to conduct enough clinical trials due to lack of research related practical training of young nutrition faculty, poor infrastructure and lack of research support systems like free access to biostatisticians and poor interdepartmental mentorship at an institutional level. As a result, very few cross-cutting high-quality landmark clinical trials have been published from these countries, despite the huge scope and need for evidence-based clinical nutrition interventions in these nations [3].

This article attempts to introduce the basic concepts of planning and executing nutrition and lifestyle interventions to early nutrition researchers and dietitians, through a very simple and step by step approach.

## Review

Nutrition and lifestyle based clinical trials: what and why?

An RCT is a quantitative, comparative and a controlled experimental study in which the researcher allocates people at random to receive one of several clinical interventions and then observes its effect on a pre-decided clinical outcome. Clinical trials are performed with a purpose of assessment of efficacy, safety, or risk benefit ratio. The goal may be superiority, non-inferiority, or equivalence [4]. Different types of trials with relevant examples are listed in Table 1.

**Table 1:** Type of trials

Type of trial (treatment groups)	Example
A new intervention to a standard one	Trial comparing an Intensive Lifestyle Intervention to Diabetes Support and Education in overweight and obese diabetes patients to assess the progression of cardiovascular disease with time [6].
A new intervention to a placebo	Double-blind, placebo-controlled RCT to compare the effect of dietary supplementation containing ginger, green tea and capsaicin on metabolic profiles and weight loss among overweight women [7].
Already existing interventions with each other	Trial to determine the effect of a healthy low-fat vs a healthy low-carbohydrate diet on weight change [8].

Nutrition and lifestyle-related trials can be very different from drug trials. Designing a lifestyle intervention trial using an optimal study design and data analysis and interpretation methods is a challenge. Lifestyle interventions are more complex in nature and require individualization as per the participants. Low rate of recruitment, high loss to follow up and issues of non-adherence are more common in nutrition and lifestyle-related trials. As compared to drug trials, these need rigorous monitoring and evaluation [5].

Are RCTs a suitable design for your clinical nutrition research question?

Before planning an RCT, a researcher should assess if the published studies suggest that the dietary intervention under question might be beneficial or not, along with checking the feasibility, ethical concerns and availability of resources to conduct the RCT. A young researcher should conduct a systematic literature search to find the available evidence before framing the research question for the RCT and also take the views of experts and mentors on the relevance of the RCT that is being planned. In scenarios where an RCT is unlikely to provide a conclusive answer, it is best to choose some other designs such as case control or cohort study designs, etc. Only when evidence from other study designs suggests that an intervention might be effective, an RCT should be planned to generate stronger evidence.

Part I: planning an RCT

It is suggested that one-third of the total time of the RCT study must be spent on detailed planning [9]. Some key points to consider while planning an RCT are:

1. Writing a Detailed Protocol Based on a Hypothesis

A detailed protocol outlining all components (research question, hypothesis, study design, primary and secondary objectives, sample size, selection of participants, outcome measures, statistical analysis) is instrumental to conduct an RCT [10]. Some checklists such as CONSORT [11] and NICE [12] are available which may act as a template to guide researchers in developing a sound protocol. Components of a good protocol include:

a) A clear title, hypothesis and objectives: The title of the RCT should be accurate, short and concise. It should preferably include all components of the PICOT (Population, Intervention, Comparator, Outcome and Time frame) format [13]. Use the SMART (Specific, Measurable, Attainable, Relevant & Time bound) criteria [14] to design a clear hypothesis. While writing objectives for a lifestyle intervention, it is very important to be sure if the research is trying to measure the “efficacy” (how the intervention performs under ideal/controlled circumstances) or the “effectiveness” (how the intervention performs under real world conditions) of the intervention. Clear demarcation of primary and secondary objectives is also important. The primary objective is the ultimate objective for which an RCT is planned and the sample size is calculated to answer the primary objective with adequate power.

b) Target population, selection criteria and sample size: Identify the target population on whom the results of the study will be generalized and a statistically significant impact of the intervention is feasible and likely. Selection criteria must 1) not be too strict 2) be aligned to the primary outcome 3) avoid possible confounding factors 4) optimize the effectiveness of active treatment 5) ease the recruitment and 6) consider the chances of non-compliance to treatment and loss to follow up [15]. Demographic details such as age and gender, duration and intensity of disease and medical history are some basic considerations while setting up the entry criteria.

For sample size calculation, it is advisable to involve a statistician to know the minimum required sample to show statistically significant results in the trial. Trials with an inadequate sample size may lead to waste of resources as well as misleading conclusions.

c) Details of control and intervention group: Include at least one control group to demonstrate that the intervention is superior/inferior/equivalent to the standard practice. A control group discriminates outcomes caused by the intervention from those caused by other factors, such as natural progression of disease, patient expectations, or other treatments [16] as both the intervention and control groups are equally matched using randomisation. A weakly designed control group may lead to misinterpretation of results in an RCT.

It is important to clearly define the intervention you want to test in the trial. In case of lifestyle interventions including modifications in dietary and exercise behaviour, it is crucial to have a standard operating procedure that clearly states the mode of administration and duration of the intervention, any run in period required, tactics to be used for recruitment and adherence to protocol, and a timeline for follow up measurements and monitoring of intervention.

d) Trial design: Trial design optimizes and economizes the trial conduct. Three commonly used trial designs for examining the effects of dietary interventions are parallel, crossover and factorial study designs.

In a parallel trial (classical clinical trial approach), one group receives only treatment A while another group receives only treatment B. The two treatment arms can be two completely separate treatments or simply different doses of the same treatment. Generally, a placebo/active control is used as control groups in parallel studies. The parallel study design allows testing multiple interventions at the same time, which leads to shorter study duration [17].

The crossover study design is carried out in two phases. In the first phase, one study arm receives treatment A and the other arm receives treatment B. The assigned treatment to each arm is interchanged in the second phase, after a washout period. Participants in crossover studies act as their own control. It is not advisable to use crossover trials in studies where the outcomes may reverse in a short time span (e.g., weight loss) or carryover for a long time (e.g., change in hepatic fat content) [18].

In a factorial study design, which is commonly used in studies with dietary supplements and nutraceuticals [19,20], each participant is randomly allocated to a combination of two or more interventions. Using a factorial study design, it is possible to evaluate the effects as well as the additive, opposite or collective interactions of multiple treatments simultaneously, using just one study. The most commonly used approach is the 2 × 2 factorial design approach involving two interventions at two levels each. It is a potentially more informative and efficient approach since it allows evaluation of multiple intervention components with good statistical power. The major concern with this study design is the interaction of interventions, which often complicates interpretation of treatment effects.

e) Precise and measurable outcomes: A limited number of clinically significant outcomes should be defined, giving details on how, when and by whom will the outcomes be measured. The outcome measures selected must be ones that can be measured accurately and precisely [21]. Continuous outcome variables that can have any value between specified intervals (for example weight, height, age) over dichotomous outcome variables (for example - Are you a vegetarian? - Yes or No) increase the power of a study, permitting a smaller sample size. Several outcome measures can be used to evaluate different aspects of the results, including the adverse effects of the intervention.

f) Methods of randomization and stratification:* *Randomization prevents selection bias and ensures that any observed differences between the treatment groups are due to differences in the treatment alone and not due to the effects of any known or unknown confounding factors. Well-designed RCTs determine the method of randomization in advance. All aspects of randomization such as type of randomization (Table 2), researcher involved in randomization, the timing of randomization and existence of a randomization register should be mentioned in the study protocol. Randomisation depends upon two important aspects; adequate generation of the allocation sequence (using computer-generated sequences, random numbers tables, drawing of envelopes) and concealment of the allocation sequence until assignment occurs using sequentially numbered, sealed, opaque envelopes.

**Table 2:** Types of Randomization

Type of Randomization	What is it?	Features	Example
Simple Randomization	Randomization based on single sequence of random assignments Example- flipping a coin	Simple and easy to implement Results can be problematic in relatively small sample clinical research as it may lead to unequal number of participants in both the groups	Toss of coin or roll of dice
Block Randomization	Randomization done to select and divide participants into different groups or conditions in order to avoid selection bias	Suitable for smaller trials to ensure equal numbers in each group	Block randomization of two treatment groups A and B Number of blocks = 3, size of blocks = 10, and fixed size blocks. BLOCK 1- 1: A 2: B 3: B 4: A 5: A 6: B 7: B 8: A 9: B 10: A BLOCK 2- 1: A 2: B 3: B 4: B 5: A 6: B 7: A 8: A 9: B 10: A BLOCK 3- 1: A 2: B 3: B 4: A 5: B 6: A 7: B 8: A 9: A 10: B
Stratified Randomization	Randomization that involves the division of a population into smaller sub-groups known as strata. The strata are formed based on members' shared attributes or characteristics such as income or gender	Ensures that a potential baseline confounding variable is equally distributed between the two groups.	In case of assessing results of a weight loss intervention on patients, stratification can be done on the basis of Body Mass Index (BMI), education status, gender, socio economic status etc. After stratification, simple randomization is applied to each stratum to assign subjects to either group.

g) Blinding: Blinding is done to hide the critical information on treatment allocation from patients, investigators or the evaluator in the study. This ensures that there are no differences in the ways in which each group is assessed or managed, and therefore bias is minimized [22]. The term “double blinding” is used when both the investigator and the study participants are not aware of treatment assignments. However, double blinding is not feasible in many nutrition and lifestyle trials such as the Look AHEAD trial of weight loss for the prevention of cardiovascular disease in type 2 diabetes [6]. Blinding of the study participants, their families and researchers involved in outcome assessment is not easy in lifestyle interventions because of active involvement of the participants in the intervention. For example, if a participant has been put on the Mediterranean diet along with a specific exercise regimen, they would know that they have been subjected to that intervention. The best possible option at times is to not tell the participants which is the active treatment arm and which is the comparator arm.

h) Plan for statistical analysis: A basic plan for statistical analysis should be formulated right at the planning stage, with the help of an experienced statistician. It should be planned whether data will be analysed on an intention-to-treat (ITT) or per-protocol (PP) basis. In ITT, every patient randomized to the study must enter primary analysis, whether or not they were compliant, early drop out or received an intervention outside of the study protocol. PP analysis only counts those patients who completed the study as specified in the protocol. PP analysis thus identifies a treatment effect that would occur under optimal conditions. The patients who choose to withdraw from the study/deviate from the protocol may differ in characteristics from those who completed the study as specified [23]. Withdrawal of the patient might be due to treatment being not effective and that is a disadvantage of per protocol. The ITT approach is generally preferred, as it maintains the advantages of randomization. 

i) Realistic timelines: The protocol must contain a schematic diagram to efficiently present the overall schedule and time points for assessment in each group of the trial. Set realistic timelines, starting from initial eligibility screening until the study closes. 

2. Getting Ethical Approval From the Institutional Review Board

Get the protocol reviewed and approved by the appropriate Ethics Committee (EC) at the institute where the study will be carried out, prior to the initiation of the trial. Details of some important considerations are listed below:

a) Informed consent: Disclosure, voluntariness, comprehension and competence are crucial aspects of an informed consent form. Informed consent has two parts: Patient Information Sheet (PIS) and Patient Informed Consent Form (PICF). The PIS must clearly indicate the nature, duration, potential benefits and risks involved in the study. The informed consent documents must be written in a language that is familiar to the participant and is easy to comprehend. Researchers must make sure that the participants have properly understood the various components of the informed consent before signing it. 

b) Clinical trial registry: All trials involving human participants, for any intervention including nutrition and lifestyle interventions, need to be registered prospectively on platforms such as the Clinical Trials Registry - India (CTRI), International Standard Randomised Controlled Trial Number Register (ISRCTN), clinicaltrials.gov, etc., before enrolment of the first participant. A registration number is allotted to the trial after registration, which is required to be reported while publication of the trial in any reputed journal. This open access registry allows the research community as well as the study participants to refer to the trial methodology when required. Some medical journals publish RCT protocols also to ensure that no modifications are done in the protocol according to trial results [24].

c) Insurance of the trial: The study protocol should contain a finance and insurance section that provides details of insurance coverage for treatment and compensation of trial-related injuries [25]. All intervention studies require an adequate insurance policy that can protect the investigator and patient from any harm resulting from participation in the clinical study.

PART II: conducting an RCT

The trial should be done absolutely in line with the protocol. Participants in both arms should be treated exactly the same way except for the intervention/control treatment, making sure that no undue testing is done on the patients in the trial.

a) Collection of baseline measurements: Use well-established tests/instruments to measure all variables. It is best to collect data as continuous variables, wherever possible. Focus should be on parameters that have the potential to objectively show the influence of intervention on the outcomes. The researcher who collects the outcome data should be blinded to the treatment allocation of the patients, to reduce the risk of bias while collecting the data.

b) Recruitment of participants: Randomize each study participant to the different treatment groups, after opening the sequentially labelled, sealed and opaque envelopes. The principles of confidentiality should be observed, and such recordings/related documentation should be preserved.

c) Follow-up and outcome assessment: Both the treatment and control group must be followed up as per the demand of the protocol, with assessment of outcomes in accordance with the time points and schedule set at the time of protocol development. The challenge here lies in reducing the loss to follow up and making sure complete data is taken from all participants without missing any important data. To reduce loss to follow up, it is important to keep the intervention easy and keep the study visits comfortable and well organised for participants. Also, select subjects who have better chances for adherence to the intervention. Plan screening visits before randomization to exclude participants who would not be able to complete the required visits [26]. Try to establish good rapport and frequent contact with subjects to maintain good adherence and reduce loss to follow up. Prefer non-invasive methods for data collection as far as possible. Test results that are of interest to the participants should be provided to them along with required interpretation and appropriate counselling.

d) Monitoring the trial:This step ensures quality control and helps maintain ethical standards. All staff involved in the trial must be monitored, trained and motivated regularly to ensure that study procedures and data handling is being carried out as planned in the protocol. The consumption of nutraceuticals, dietary supplements or a particular diet may cause adverse effects that should be adequately monitored and investigated. A group of clinicians and biostatisticians form the data monitoring committee in a clinical trial. They are appointed by study sponsors to provide independent assessment on the validity, safety and integrity of the trial. Such committees are generally needed in trials that assess novel interventions and can have a major impact on clinical practice [27].

A number of challenges associated with the planning and monitoring of RCTs and their possible solutions are summarised in Table 3.

**Table 3:** How to tackle the challenges associated with conducting RCTs? RCT: randomized controlled trial

S. No	Challenge	Possible solutions
1	Unclear hypothesis and multiple objectives	Use the PICOT method (Population, Intervention, Comparison, Outcome and Timeframe) to state your research hypothesis. Avoid having too many objectives in a trial. Opt for one primary and few secondary outcomes.
2	Inefficient entry criteria	Balance the pros and cons of selecting very strict versus very lenient selection criteria.
3	Irrelevant and non-significant interventions	Choose interventions that would be feasible and practical in relevant clinical settings.
4	Ineffective randomization, stratification and blinding	Record and log all methods of randomization and randomization attempts. Stratify to prevent imbalances especially in small studies. Always blind outcome assessors to treatment allocation.
5	Insufficient sample size	Involve an expert statistician since the planning phase of the trial to avoid mistakes in sample size calculation.
6	Low recruitment and loss to follow up	Account for refusal to consent in the beginning. Anticipate crossover between control and intervention arm, loss to follow up and recruitment rate of below 50%, to maintain power of the study.
7	Failure to use Intention to treat analysis	Everyone who begins the treatment should be considered part of the trial to avoid misleading biases.
8	Quality control	Use a standardized operations manual for all procedures. Develop user-friendly and clearly formatted data collection forms, focusing only on relevant data.

e) Interim analysis: Pre-planned interim analysis by independent statisticians is helpful in early assessment of efficacy as well as safety of the nutrition/lifestyle interventions. It can be described as the evaluation of trial data before the recruitment is complete to allow for any changes to be made in the ongoing trial. Standard operating practices, research integrity, regulatory concerns and scientific reasoning must be maintained while planning to conduct an interim analysis.

f) Analysis and interpretation: Statistical analysis should be carried out as planned in the protocol. Estimate the intervention’s effect on all pre-decided study outcomes. Report the statistical significance and magnitude of effect on the outcomes. It is crucial to report the statistical tests to show no difference in key characteristics between both groups at the baseline. Analysis should be done to check whether loss to follow up created differences between the two groups during the intervention period. 

## Conclusions

To summarize, RCTs are the gold standard in evidence-based nutrition practice to establish causal relations between exposure to diet/nutrients and pre-decided outcome measures, such as anthropometric or biochemical parameters. This review aims to educate young nutrition graduates and dietitians about the basics of planning and executing trials to claim the efficacy/effectiveness of nutrition interventions, while maintaining ethical and scientific integrity. Addressed in this document are specific aspects of planning (drafting a research question, creating a hypothesis, determining primary and secondary objectives, selection of participants, randomization, blinding, deciding outcome measures and statistical analysis) and conducting (recruitment of participants, data collection, follow ups and outcome assessment, monitoring of the trial, analysis and interpretation of results) a nutrition-based trial, along with focus on additional important topics such as ethical considerations, clinical trial registry and trial insurance. 
